# Multisystem inflammatory syndrome in children during the COVID-19 waves: data from the Juvenile Inflammatory Rheumatism cohort

**DOI:** 10.3389/fped.2023.1126985

**Published:** 2023-05-24

**Authors:** Robin Kechiche, Charlotte Borocco, Fanny Bajolle, Alexandre Belot, Sylvaine Poignant, Noémie Lachaume, Lucas Percheron, Ulrich Meinzer, Clara Mertes, Véronique Despert, Luc Morin, Virginie Lambert, Perrine Dusser, Nassima Matsa, Véronique Hentgen, Isabelle Kone-Paut, Caroline Galeotti

**Affiliations:** ^1^ Department of Pediatric Rheumatology, Reference Centre for Autoinflammatory Diseases and Amyloidosis (CEREMAIA), Bicêtre University Hospital, Assistance Publique-Hôpitaux de Paris (AP-HP), Paris-Saclay University, Le Kremlin-Bicêtre, France; ^2^ Department of Pediatric Cardiology, M3C-Necker, Necker-Enfants Malades Hospital, AP-HP, Paris Cité University, Paris, France; ^3^ Department of Pediatric Nephrology, Rheumatology, Dermatology, Reference Centre of Inflammatory Rheumatism and Rare Autoimmune Diseases in Children (RAISE), Hôpital Femme-Mère-Enfant, Hospices Civils de Lyon, Bron, France; ^4^ Department of Pediatrics, Nantes University Hospital, Nantes, France; ^5^ Department of Pediatrics, Louis Mourier University Hospital, AP-HP, Paris-Cité University, Colombe, France; ^6^ Pediatrics—Nephrology, Internal Medicine and Hypertension, Children Hospital—Toulouse University Hospital, Toulouse, France; ^7^ Department of General Pediatrics, Pediatric Rheumatology and Infectious Diseases, National Reference Centre for Rare Pediatric Inflammatory Rheumatisms and Systemic Autoimmune Diseases (RAISE), Robert Debré University Hospital, Assistance Publique-Hôpitaux de Paris, Paris, France; ^8^ Department of Pediatrics, Strasbourg University Hospital, Strasbourg, France; ^9^ Department of Pediatrics, Rennes University Hospital, Rennes, France; ^10^ Pediatric Intensive Care Unit, Bicêtre University Hospital, AP-HP, Paris-Saclay University, Le Kremlin-Bicêtre, France; ^11^ Department of Pediatric Radiology, Bicêtre University Hospital, AP-HP, Paris-Saclay University, Le Kremlin-Bicêtre, France; ^12^ Pediatric Cardiology, Institut Mutualiste Montsouris, Paris, France; ^13^ Department of Pediatrics, Reference Centre for Autoinflammatory Diseases and Amyloidosis (CEREMAIA), Versailles Hospital, Versailles, France

**Keywords:** multisystem inflammatory syndrome in children, COVID-19, waves of COVID-19, epidemiology, myocarditis

## Abstract

**Introduction:**

Multisystem inflammatory syndrome in children (MIS-C) is a new condition that first appeared in children and adolescents during the COVID-19 pandemic. We aimed to describe the diagnostic course, clinical and biological manifestations, and treatment of MIS-C during the first three COVID-19 waves.

**Methods:**

We extracted patient data from the Juvenile Inflammatory Rheumatism (JIR) cohort. We analyzed data for patients meeting the World Health Organization diagnostic criteria for MIS-C from the start of the COVID-19 pandemic from March 2020 to June 30, 2021. We then compared data for patients in wave one to those in waves two and three.

**Results:**

We identified 136 patients with MIS-C. The median age decreased but not significantly during the waves, from 9.9 years to 7.3 years (*p* = 0.105). Boys represented 52.2% (*n* = 71) of patients, and 46% (*n* = 41) of patients originated from sub-Saharan Africa (*p* < 0.001). Patients presented less diarrhea (*p* = 0.004), respiratory distress (*p* < 0.001), and myocarditis (*p* < 0.001) with progressive waves. Biological inflammation also decreased, namely, C-reactive protein level (*p* < 0.001), neutrophil count (*p* = 0.004), and albumin level (*p* < 0.001). Patients received more corticosteroids (*p* < 0.001) and required less ventilation support (*p* < 0.01) and less inotrope treatment (*p* < 0.001) in the later waves. The duration of hospitalization gradually decreased (*p* < 0.001), as did critical care unit admissions (*p* = 0.002).

**Conclusion:**

Over the three COVID-19 waves, with a change in the management of MIS-C, children in the JIR cohort in France showed a less severe disease course, in particular, a greater use of corticosteroids. This observation may reflect the impact of both improved management and different SARS-CoV-2 variant.

## 1. Introduction

Pediatric patients infected with SARS-CoV-2 (COVID-19) represented a very small proportion of hospitalizations for COVID-19 during the pandemic (1). However, in March 2020, a new SARS-CoV-2–related hyper-inflammatory phenotype multisystem inflammatory syndrome in children (MIS-C)/pediatric inflammatory multisystem syndrome (PIMS) arose in children and adolescents living in Europe, the United States, and then the rest of the world (2–5). The underlying mechanisms of MIS-C/PIMS are still unclear, but the syndrome has the profile of an immunological disease that develops at a distance from the primary infection with SARS-CoV-2. The clinical phenotype is close to toxic shock syndrome, and an amplified restricted Vβ T-lymphocyte response strongly suggests a superantigen type of the immunological response (6). The international WHO criteria for MIS-C were defined in May 2020 (7). However, all the early signs are not very specific and can mislead the diagnosis by delaying appropriate treatment.

The first patients with MIS-C were admitted to intensive care units with acute heart failure variably associated with some mucocutaneous signs reminiscent of Kawasaki disease. Therefore, in addition to inotropic drugs, immunomodulatory therapies including intravenous immunoglobulins (IVIg), aspirin, and corticosteroids were used. With the succeeding COVID-19 waves, the description of MIS-C became more refined, and guidelines for its management were established (8). On December 1, 2020, the French MIS-C task force proposed the systematic use of corticosteroids for MIS-C. Concomitantly, since the beginning of the pandemic, SARS-CoV-2 variants have circulated, possibly modifying the presentation, severity, and evolution of MIS-C.

In this context, we compared the diagnostic course and clinical and biological manifestations of MIS-C and the therapeutic strategies for cases reported in France. We used medical data for the Juvenile Inflammatory Rheumatism (JIR) cohort during the first three waves of COVID-19 between March 2020 and the end of June 2021. In addition, we analyzed the association between the duration of hospitalization and clinical and biological data and looked for potential factors associated with cardiac dysfunction.

## 2. Methods

### 2.1. Data source

The JIR cohort (https://www.jircohorte.org) is an international prospective and retrospective cohort of patients with pediatric inflammatory rheumatism. French centers prospectively recorded patients with a diagnosis of MIS-C from March 2020. The database includes demographic data, vital sign measurements, and diagnostic and treatment information for all patients.

### 2.2. Ethical considerations

This study was compliant with the principles of the Declaration of Helsinki, and the protocol was approved by the French ethics committee (CCTIRS) on April 21, 2015. Patients were recruited after information and verification that they (or their legal guardians) did not object to the study and the storage of their personal data. The electronic case report form was approved by the *Commission Nationale de l'Informatique et des Libertés* (CNIL).

### 2.3. Study population

**Figure 1:**
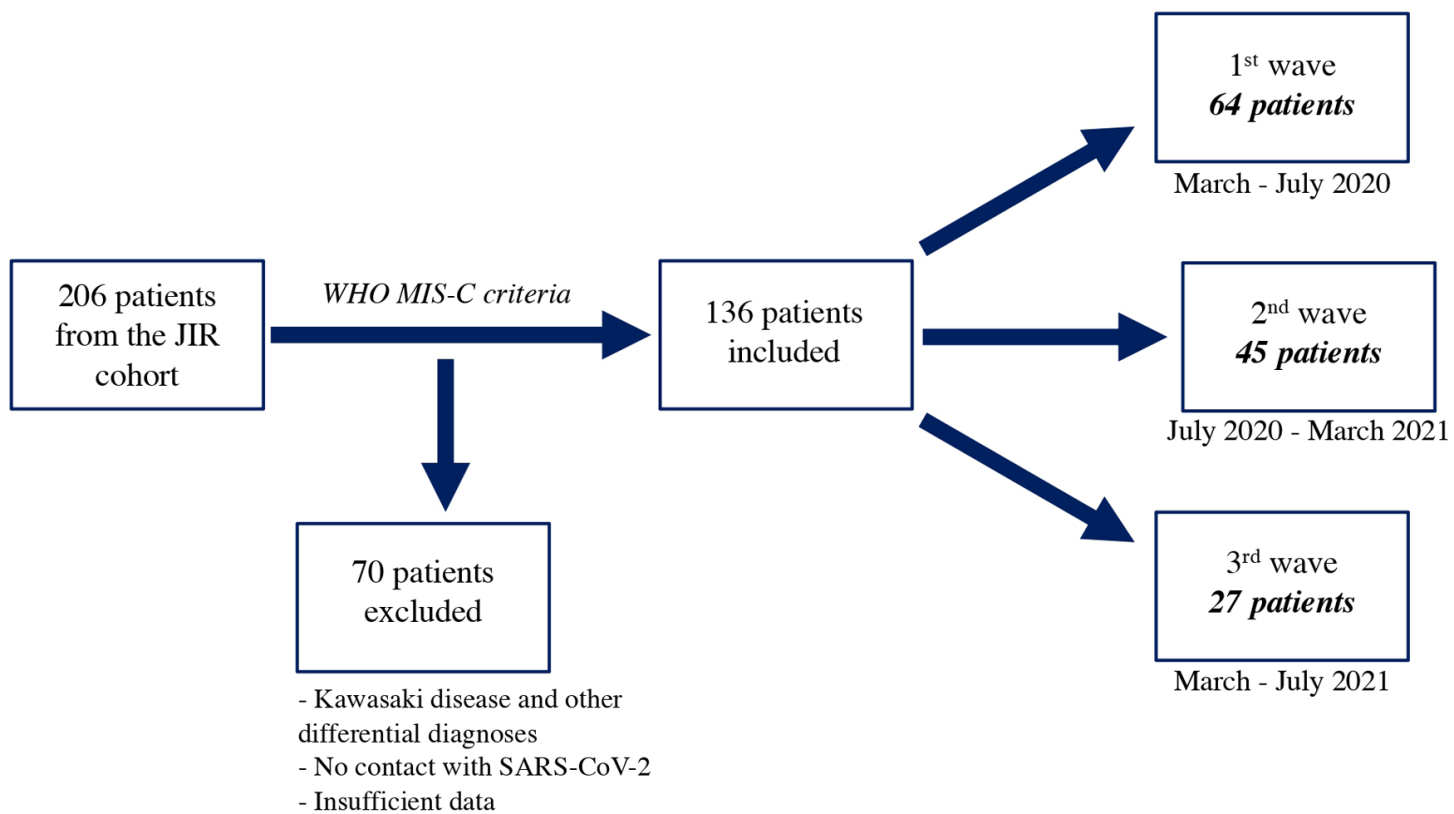
Flow chart of selection of patients.

All patients aged 0–18 years who were included in the JIR cohort and had a diagnosis of MIS-C between March 2, 2020, and June 30, 2021, according to the WHO criteria were considered. A total of 206 patients were identified from 10 different centers. We excluded 70 patients because of a diagnosis of typical Kawasaki disease and lack of evidence of previous SARS-CoV-2 infection. We also excluded patients with more than 20% missing values (Figure 1).

**Figure 2:**
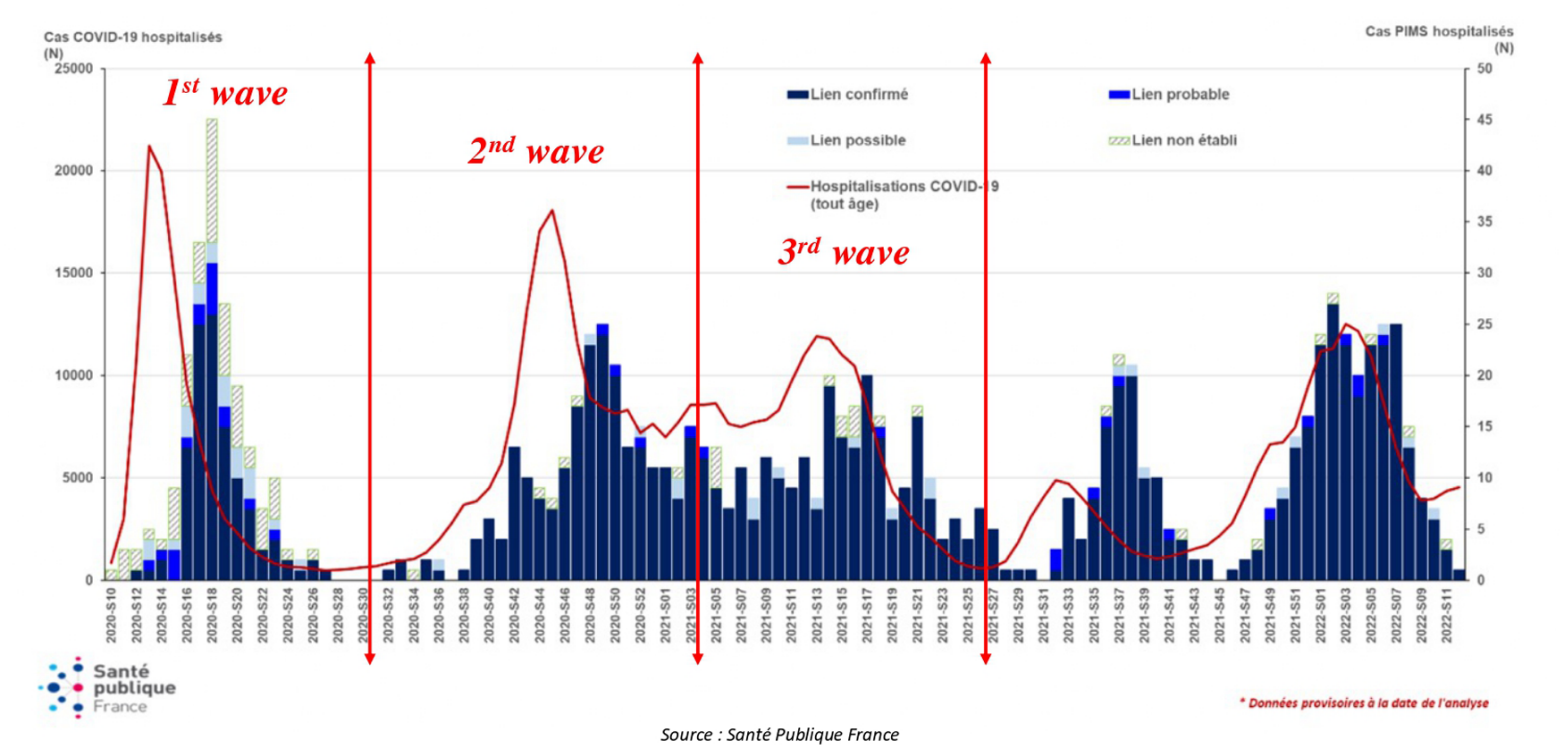
Selection of patients. Weekly number of hospital admissions for MIS-C according to link with COVID-19 and COVID-19 hospital admissions for all ages from March 2, 2020, to February 20, 2022. (Source: Santé Publique France). MIS-C, multisystem inflammatory syndrome in children. This figure is adapted from a Santé publique France chart.

We divided patients into three groups according to the date of MIS-C occurrence and in accordance with the temporal division of the waves of COVID-19 defined by the Santé Publique France. The first group corresponded to the period from March 2, 2020 (week 10), to July 26, 2020 (week 30), determined as the first wave of COVID-19. The second group corresponded to the period from July 27, 2020 (week 31), to January 24, 2021 (week 3), and the third group to the period from January 25, 2021 (week 4), to June 30, 2021 (week 26), determined as the second and third waves (Figure 2).

### 2.4. Measures

Information collected included patient demographics, clinical manifestations, laboratory test results, imaging studies, and management (inpatient unit, treatments). Myocardial dysfunction was defined as a left ventricular ejection fraction <60%.

Differential diagnoses and associated diagnoses were recorded, as were all treatments administered. The time to administration of corticosteroids was analyzed. It was defined as the time in days from the date of onset of symptoms to the date of initiation of corticosteroids. The time to corticosteroid administration could be calculated for about 93% of the patients who received this therapy (90 of 97 patients).

### 2.5. Statistical analysis

Descriptive statistics are described with numbers (%) for categorical variables and median [interquartile range (IQR)] for quantitative variables. To evaluate the association of characteristics between the three groups, we used ANOVA for quantitative variables and the Fisher test or *χ*^2^ test for qualitative and categorical variables. *p* < 0.05 was considered statistically significant. Spearman's correlation coefficient was calculated to describe the correlation between hospitalization duration and quantitative biological and clinical data. We then used a multivariate logistic regression model to identify factors associated with cardiac dysfunction, estimating odds ratios (ORs) and 95% confidence intervals (CIs). Variables significant at *p* ≤ 0.25 on univariate analyses were included in the multivariate model by stepwise selection. All statistical analysis and calculations were performed with R 4.1.3 (R Core Team).

### 2.6. Missing data handling

Variables with missing data are a common occurrence in databases. However, analyses that ignore missing data have the potential to produce biased results. Therefore, we used multiple imputations to deal with missing data. All selected variables had <20% missing values. Data were assumed missing at random and were imputed by using fully conditional specification with the R 4.1.3 “mice” package (v 3.14.0).

## 3. Results

### 3.1. Description of the cohort

#### 3.1.1. Demographic characteristics

We included 136 patients aged 0–18 years; 64 had a diagnosis of MIS-C during the first COVID-19 wave, 45 during the second wave, and 27 during the third wave from 10 different hospital centers. The median age was 8.5 years (IQR: 4.9–11.6) and 65 (47.8%) were girls. Overall, 33 (32.7%) patients were obese or overweight according to the body weight curves of the National Nutrition and Health Plan. In the whole cohort, 46% of patients originated from sub-Saharan Africa and 27% from the Mediterranean region, and 18% were Western Caucasians (Table 1).

**Table 1:** Epidemiological data for cases of multisystem inflammatory syndrome in children (MIS-C) by COVID-19 waves in France.

		COVID-19 waves			
	Total	First wave	Second wave	Third wave	*p*-value
Sex, *n* (%)	*n* = 136	*n* = 64	*n* = 45	*n* = 27	**0** **.** **013**
Male	71 (52.2)	26 (40.6)	31 (68.9)	14 (51.9)	
Female	65 (47.8)	38 (59.4)	14 (31.1)	13 (48.1)	
Age (years), *n* (%)	*n* = 136	*n* = 64	*n* = 45	*n* = 27	0.083
≤2	9 (6.6)	4 (6.2)	1 (2.2)	4 (14.8)	
2–5	27 (19.9)	9 (14.1)	14 (31.1)	4 (14.8)	
5–12	69 (50.7)	32 (50)	24 (53.3)	13 (48.1)	
>12	31 (22.8)	19 (29.7)	6 (13.3)	6 (22.2)	
Median (IQR)	8.5 (4.9–11.6)	9.9 (6.2–12.4)	7.3 (4.3–10.3)	7.7 (4.9–10.6)	0.105
Ethnicity, *n* (%)	*n* = 89	*n* = 46	*n* = 23	*n* = 20	**<0** **.** **001**
Sub-Saharan African	41 (46)	30 (65.2)	6 (26.1)	5 (25)	
Mediterranean	24 (27)	7 (15.2)	10 (43.5)	7 (35)	
Caucasian	16 (18)	3 (6.5)	6 (26.1)	7 (35)	
Caribbean	3 (3.4)	2 (4.3)	1 (4.3)	0 (0)	
Mixed	3 (3.4)	3 (6.5)	0 (0)	0 (0)	
Asian	1 (1.1)	1 (2.2)	0 (0)	0 (0)	
North American	1 (1.1)	0 (0)	0 (0)	1 (5)	
Comorbidity, *n* (%)	*n* = 136	*n* = 64	*n* = 45	*n* = 27	
Hypertension	0 (0)	0 (0)	0 (0)	0 (0)	
Diabetes	0 (0)	0 (0)	0 (0)	0 (0)	
Renal insufficiency	0 (0)	0 (0)	0 (0)	0 (0)	
Sickle cell disease	2 (1.5)	2 (3.1)	0 (0)	0 (0)	
Heart disease	1 (0.7)	1 (1.6)	0 (0)	0 (0)	
Other	4 (2.9)	3 (4.7)	1 (2.2)	0 (0)	
BMI, *n* (%)	*n* = 101	*n* = 52	*n* = 27	*n* = 22	
Underweight	2 (2)	1 (1.9)	0 (0)	1 (4.5)	
Normal	66 (65.3)	37 (71.2)	16 (59.3)	13 (59.1)	
Overweight	18 (17.8)	8 (15.4)	5 (18.5)	5 (22.7)	
Obesity	15 (14.9)	6 (11.5)	6 (22.2)	3 (13.6)	
Overweight or obesity	33 (32.7)	14 (26.9)	11 (40.7)	8 (36.4)	
Mean (SD) kg/m^2^	19.4 (4.4)	19.4 (4.6)	19.1 (4.1)	19.7 (4.6)	0.913

MIS-C, multisystem inflammatory syndrome in children; IQR, interquartile range.

Bold values mean significant *p* < 0.05.

#### 3.1.2. Clinical, biological, and imaging characteristics

The main symptoms of MIS-C were fever (98.5%), rash (68.9%), abdominal pain (65.9%), symptoms of myocarditis (65.9%), conjunctivitis (65.2%), lip cracking (53%), diarrhea (50%), vomiting (47%), headache (43.2%), pharyngitis (34.1%), respiratory distress (33.3%), cervical adenopathy (26.5%), and pericarditis (20.5%) (Table 2).

**Table 2:** Comparison of symptoms.

		COVID-19 waves			
	Total	First wave	Second wave	Third wave	*p*-value
General, *n* (%)	*n* = 132	*n* = 60	*n* = 45	*n* = 27	
Fever	130 (98.5)	58 (96.7)	45 (100)	27 (100)	0.203
Digestive, *n* (%)	* *	* *	* *		
Abdominal pain	87 (65.9)	45 (75)	28 (62.2)	14 (51.9)	0.088
Diarrhea	66 (50)	38 (63.3)	14 (31.1)	14 (51.9)	**0.004**
Vomiting	62 (47)	33 (55)	21 (46.7)	8 (29.6)	0.085
Respiratory, *n* (%)	* *	* *	* *	* *	* *
Respiratory distress	44 (33.3)	34 (56.7)	7 (15.6)	3 (11.1)	**<0** **.** **001**
Cough	19 (14.4)	9 (15)	7 (15.6)	3 (11.1)	0.853
Cardiac, *n* (%)	* *	* *	* *	* *	* *
Myocarditis	87 (65.9)	51 (85)	25 (55.6)	11 (40.7)	**<0** **.** **001**
Pericarditis	27 (20.5)	16 (26.7)	7 (15.6)	4 (14.8)	0.271
Coronary dilatation	17 (12.9)	10 (16.7)	3 (6.7)	4 (14.8)	0.268
Coronary aneurysms	0 (0)	0 (0)	0 (0)	0 (0)	
Mucocutaneous, *n* (%)	* *	* *	* *	* *	* *
Rash	91 (68.9)	39 (65)	34 (75.6)	18 (66.7)	0.484
Conjunctivitis	86 (65.2)	39 (65)	30 (66.7)	17 (63)	0.950
Lip cracking	70 (53)	34 (56.7)	20 (44.4)	16 (59.3)	0.335
Palmoplantar rash	21 (15.9)	15 (25)	4 (8.9)	2 (7.4)	**0** **.** **031**
Desquamation	21 (15.9)	20 (33.3)	1 (2.2)	0 (0)	**<0** **.** **001**
Edema extremities	15 (11.4)	8 (13.3)	4 (8.9)	3 (11.1)	0.773
Neurological, *n* (%)	* *	* *	* *	* *	* *
Headache	57 (43.2)	28 (46.7)	20 (44.4)	9 (33.3)	0.492
Meningeal syndrome	30 (22.7)	19 (31.7)	6 (13.3)	5 (18.5)	0.069
ENT, *n* (%)	* *	* *	* *	* *	* *
Pharyngitis	45 (34.1)	24 (40)	11 (24.4)	10 (37.0)	0.225
Cervical adenopathy	35 (26.5)	22 (36.7)	7 (15.6)	6 (22.2)	**0** **.** **042**
Rhinorrhea	9 (6.8)	3 (5)	2 (4.4)	4 (14.8)	0.238
Osteoarticular, *n* (%)	* *	* *	* *	* *	* *
Myalgia	19 (14.4)	9 (15)	7 (15.6)	3 (11.1)	0.852
Arthralgia	4 (3)	1 (1.7)	1 (2.2)	2 (7.4)	0.400
Arthritis	0 (0)	0 (0)	0 (0)	0 (0)	

Bold values mean significant *p* < 0.05.

At diagnosis, blood tests showed marked inflammation [median maximum C-reactive protein (CRP) level 224 mg/L (IQR: 160–313), fibrinogen level 6.3 g/L (IQR: 5.3–7.9), ferritin level 540 µg/L (IQR: 288–1,036), and neutrophil count 11,390/mm^3^ (IQR: 8,690–18,100)] (Table 3). Some biological parameters favored a macrophagic activation syndrome: median lymphocyte count 900/mm^3^ (IQR: 650–1,368), moderately elevated transaminase levels [aspartate aminotransferase 62 IU/L (IQR: 36–105), alanine transaminase 52 IU/L (IQR: 25–89), triglycerides 2.4 mmol/L (IQR: 1.8–3.3), and lactate dehydrogenase 345 IU/L (IQR: 271–420)]. Median troponin and brain natriuretic peptide levels were elevated [113 ng/L (IQR: 35–417) and 3,028 ng/L (IQR: 1,309–9,437)]. Patients showed a hypercoagulable state, with a median D-dimer level of 3,540 µg/L.

**Table 3:** Complementary examinations.

		COVID-19 waves			
	Total	First wave	Second waves	Third waves	*p*-value
Laboratory tests, *median* (IQR)	*n* = 136	*n* = 64	*n* = 45	*n* = 27	* *
CRP max. (mg/L)	224 (160–313)	289 (208–352)	211 (160–289)	151 (115–205)	**<0** **.** **001**
Hematology, *median* (IQR)	*n* = 136	*n* = 64	*n* = 45	*n* = 27	* *
Lymphocytes min. (/mm^3^)	900 (650–1,368)	930 (630–1,400)	855 (640–1,310)	945 (703–1,165)	0.950
Neutrophils max. (/mm^3^)	11,390 (8,690–18,100)	14,250 (9,400–19,350)	10,110 (7,775–14,290)	10,520 (4,860–14,440)	**0.004**
Platelets min. (g/L)	166 (123–218)	174 (137–219)	154 (101–197)	153 (127–246)	0.129
Platelets max. (g/L)	468 (280–604)	539 (422–651)	305 (227–494)	375 (235–535)	**<0** **.** **001**
Hemostasis, *median* (IQR)	*n* = 136	*n* = 64	*n* = 45	*n* = 27	* *
Fibrinogen max. (g/L)	6.3 (5.3–7.9)	6.2 (5.1–7.9)	6.3 (5.6–7.4)	6.3 (5.3–6.8)	0.396
D-dimer max. (µg/L)	3,540 (1,953–5,105)	3,895 (2,063–5,705)	3,199 (2,231–5,126)	2,379 (1,630–3,830)	0.152
Biochemistry, *median* (IQR)	*n* = 136	*n* = 64	*n* = 45	*n* = 27	* *
Ferritin max. (µg/L)	540 (288–1,036)	814 (300–1,282)	540 (252–740)	368 (283–713)	0.559
AST max. (IU/L)	62 (36–105)	72 (47–117)	44 (31–66)	47 (31–79)	0.091
ALT max. (IU/L)	52 (25–89)	64 (29–101)	44 (21–75)	34 (24–86)	0.145
Triglycerides max. (mmol/L)	2.4 (1.8–3.3)	2.3 (1.7–3.9)	2.5 (2.4–2.7)	2.5 (2.2–2.9)	0.879
Albumin min. (g/L)	23 (20–28)	21 (19–24)	27 (23–31)	30 (27–33)	**<0** **.** **001**
Natremia min. (mmol/L)	130 (128–133)	130 (128–133)	132 (129–134)	132 (129–133)	0.324
Creatine kinase max. (IU/L)	129 (50–309)	153 (58–309)	81 (43–267)	48 (N/A)	N/A
LDH max. (IU/L)	345 (271–420)	359 (277–425)	254 (212–327)	410 (N/A)	N/A
Cardiac markers, *median* (IQR)	*n* = 136	*n* = 64	*n* = 45	*n* = 27	* *
Troponin max. (ng/L)	113 (35–417)	273 (66–560)	89 (27–195)	84 (23–171)	0.515
BNP max. (ng/L)	3,028 (1,309–9,437)	2,231 (570–7,353)	4,497 (2,283–9,110)	3,954 (2,020–10,279)	0.361
Other, *n* (%)	*n* = 86	*n* = 40	*n* = 33	*n* = 13	
Macrophage activation syndrome	22 (25.6)	14 (35)	6 (18.2)	2 (15.4)	0.169
Microbiology, *n* (%)	*n* = 136	*n* = 64	*n* = 45	*n* = 27	* *
Positive nasoph. COVID-19 PCR	41 (30.1)	24 (37.5)	9 (20)	8 (29.6)	0.138
	*n* = 133	*n* = 61	*n* = 45	*n* = 27	
COVID-19 positive serology	129 (97)	60 (98.4)	43 (95.6)	26 (96.3)	0.675
	*n* = 61	*n* = 46	*n* = 12	*n* = 3	
Positive blood culture	3 (4.9)	3 (6.5)	0 (0)	0 (0)	0.418
	*n* = 53	*n* = 37	*n* = 12	*n* = 4	
Leukocyturia	31 (58.5)	24 (64.9)	6 (50)	1 (25)	0.243
	*n* = 37	*n* = 28	*n* = 7	*n* = 2	* *
Active EBV replication	15 (40.5)	9 (32.1)	5 (71.4)	1 (50)	0.161
Imaging, *n* (%)	*n* = 68	*n* = 49	*n* = 15	*n* = 4	* *
Abnormal chest x-ray	40 (58.8)	33 (67.3)	5 (33.3)	2 (50)	0.061
	*n* = 110	*n* = 61	*n* = 36	*n* = 13	
Abnormal cardiac US	70 (63.6)	41 (67.2)	19 (52.8)	10 (76.9)	0.204
	*n* = 32	*n* = 28	*n* = 3	*n* = 1	
Abnormal chest CT	18 (56.3)	17 (60.7)	1 (33.3)	0 (0)	0.284
	*n* = 36	*n* = 27	*n* = 7	*n* = 2	
Abnormal cardiac MRI	7 (19.4)	6 (22.2)	1 (14.3)	0 (0)	0.571
Technical procedure, *n* (%)	*n* = 136	*n* = 64	*n* = 45	*n* = 27	* *
Lumbar puncture	21 (15.4)	16 (25)	3 (6.7)	2 (7.4)	**0.013**

CRP, C-reactive protein; AST, aspartate aminotransferase; ALT, alanine transaminase; N/A, not applicable; LDH, lactate dehydrogenase; BNP, brain natriuretic protein; EBV, Epstein–Barr virus; US, ultrasound; CT, computed tomography; MRI, magnetic resonance imaging.

Bold values mean significant *p* < 0.05.

A total of 70 (63.6%) patients had myocardial dysfunction (reduced left ventricular ejection fraction on ultrasonography), 40 (58.8%) had pulmonary abnormalities on chest radiography, and 18 (56.3%) had abnormalities on chest CT. Overall, 36 patients underwent cardiac MRI, at a median of 22.5 days (IQR: 19–25.8) after symptom onset, and 19.4% of cases (*n* = 7) were described as abnormal. MRIs for two cases in the first group met the Lake Louise criteria (diagnostic criteria of acute myocarditis), and none in the second and third groups.

In this study, seven patients with neck and throat pain in a febrile setting underwent cervical CT scans. For two patients, an aspect of retropharyngeal infiltration was described. Non-compressive adenopathy was reported in six patients.

#### 3.1.3. Differential diagnoses

In addition to Kawasaki disease, 111 differential diagnoses were evoked in patients with MIS-C. The most frequent were digestive surgical pathologies (*n* = 36 patients), mesenteric adenopathy (*n* = 23), meningeal syndrome (*n* = 21), septic shock (*n* = 9), and febrile torticollis (*n* = 7). All patients with torticollis underwent a neck CT scan, and two patients had ENT/pharyngeal complications. Four of these patients received targeted antibiotic therapies. Other less-frequent differential diagnoses were toxic shock syndrome (*n* = 4 patients); vasculitis (*n* = 3); herpetic encephalitis (*n* = 2); thoracic myelitis; cervical spondylodiscitis; lupus; hand, foot, and mouth syndrome; scarlet fever; and mycoplasma infection.

#### 3.1.4. Associated diagnoses

Associated diagnoses were kidney damage (renal failure or tubulopathy) (*n* = 51), systemic lung disease (*n* = 9), acute pancreatitis (*n* = 5), pulmonary embolism (*n* = 2), *Escherichia coli* pyelonephritis (*n* = 2), and macrophage activation syndrome (MAS; *n* = 22). The biological definition of MAS was based on the criteria of Ravelli et al. (9), established in patients with systemic juvenile idiopathic arthritis. These criteria were ferritin level >684 µg/L and at least two of platelet count ≤181 G/L, aspartate aminotransferase level >48 IU/L, triglycerides level >1.76 mmol/L, and fibrinogen level ≤3.6 g/L. The classification was possible for only patients with available ferritin assay results and results for at least two assays among platelet count and aspartate aminotransferase, triglycerides, and fibrinogen levels, that is, 86 patients (40, 33, and 13 in the first, second, and third groups, respectively). Thus, 25.6% MAS cases were observed: 35%, 18.2%, and 15.4% in the first, second, and third waves, respectively, with no significant difference (*p* = 0.169).

#### 3.1.5. Management

The patients’ first unit of hospitalization was general pediatrics (37%), intensive care unit (34.6%), intermediate care unit (12.6%), and pediatric rheumatology (8.7%) (*n* = 127); more than half of the patients (*n* = 97, 76.4%) were admitted to the intensive care unit during their hospitalization. The median time to diagnosis was 5 days (IQR: 3–6), and the median hospitalization duration was 8 days (IQR: 6–11).

**Figure 3:**
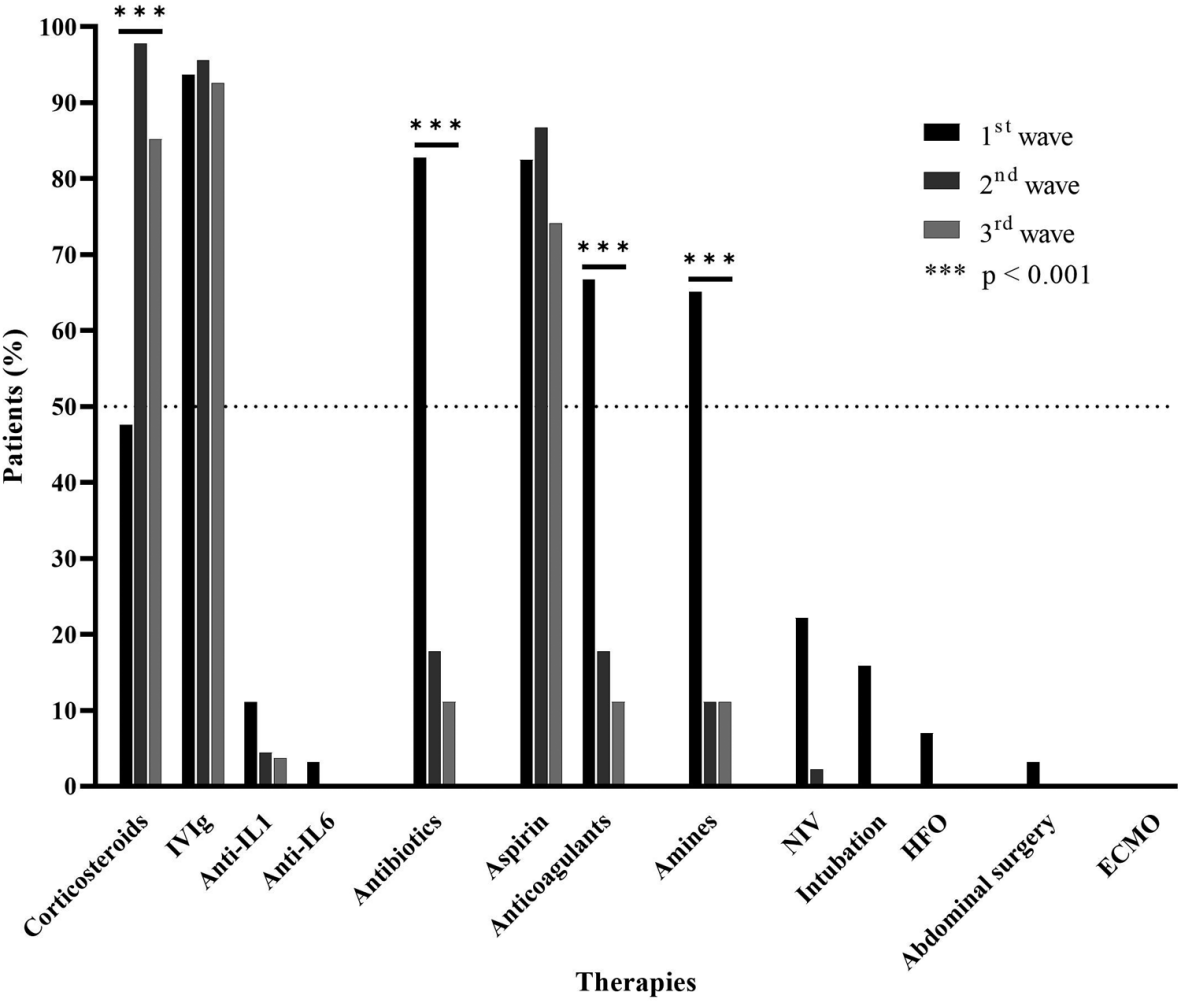
Therapies (%). IVIg, intravenous immunoglobulins; NIV, noninvasive ventilation; HFO, high-flow oxygen; ECMO, extracorporeal membrane oxygenation.

A total of 127 (93.3%) patients received IVIg therapy and 97 (71.3%) received corticosteroids. Also, 64 patients (47%) received antibiotics, 111 (81.6%) aspirin, 53 (38.9%) anticoagulants, and 49 (36%) inotropes (*n* = 136) (Figure 3). Furthermore, 10 patients received anti-interleukin-1 (anti-IL1) drugs and two anti-IL6 drugs. Finally, 32 patients (23.5%) required ventilatory support: 15 (11%) noninvasive ventilation, 7 (5.1%) high-flow nasal cannula, and 10 (7.3%) invasive ventilation. No patient required extracorporeal oxygenation, and none received anti-tumor necrosis factor alpha biotherapy.

### 3.2. Comparison of the three groups of multisystem inflammatory syndrome in children

The three MIS-C groups differed but not significantly in the median age (9.9 years in the first group vs. 7.3 in the second group vs. 7.7 in the third group; *p* = 0.105) (Table 1). The median hospitalization duration differed (9 vs. 8 vs. 5 days (*p* < 0.001), especially the intensive care unit duration (84.7% vs. 75.6% vs. 48.1%; *p* = 0.002). Furthermore, the mean diagnosis time was higher in the first and second groups than that in the third group: 8.8 vs. 6.3 vs. 4.6 days (*p* = 0.288).

**Figure 4:**
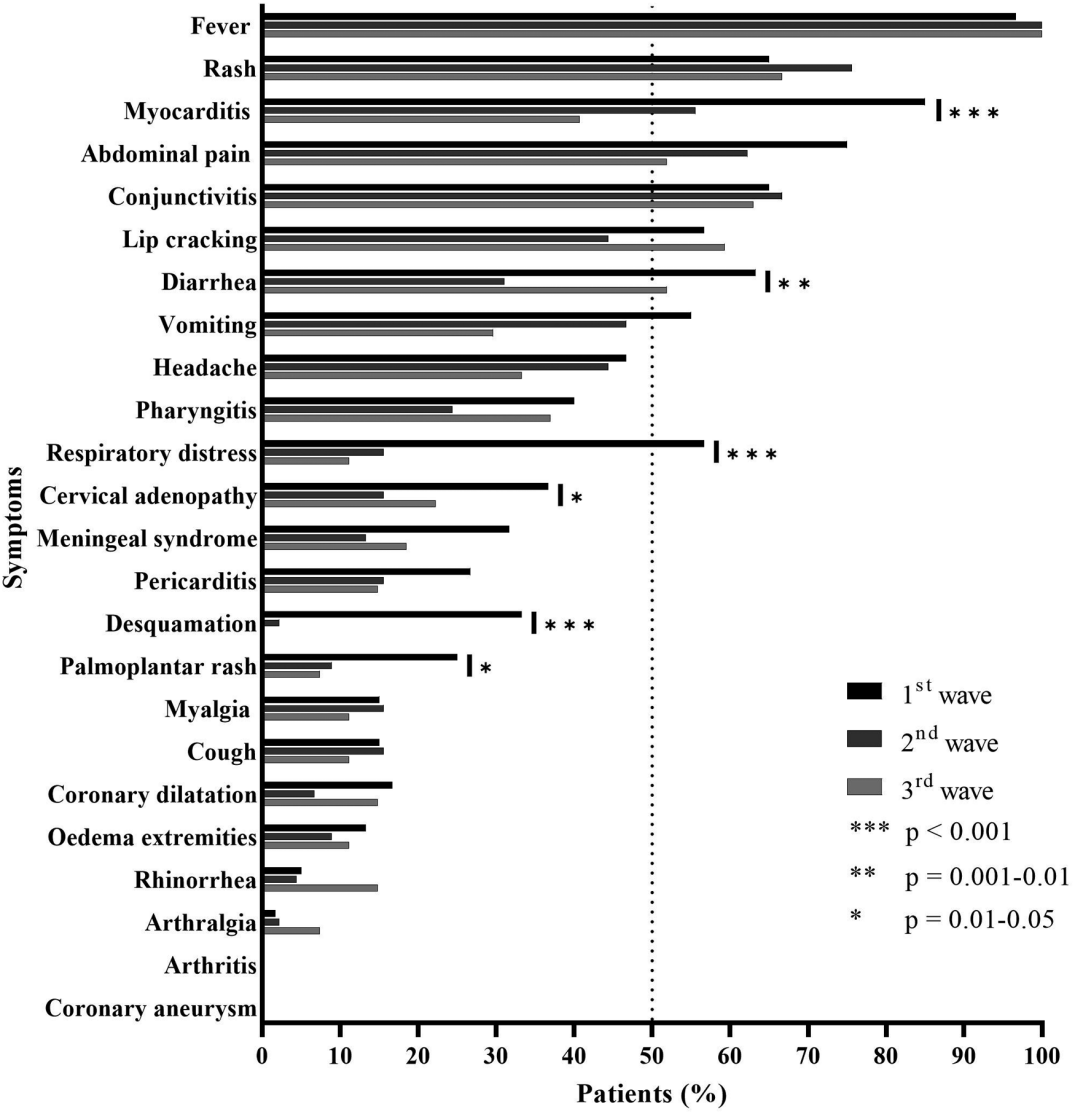
Patient symptoms (%).

At the presentation to the emergency department, diarrhea was more frequent in the first group than that in the second and third groups (63.3% vs. 31.1% vs. 51.9%; *p* = 0.004), as palmoplantar rash (25% vs. 8.9% vs. 7.4%; *p* = 0.031), desquamation of extremities (33.3% vs. 2.2% vs. 0%; *p* < 0.001), and cervical adenopathy (36.7% vs. 15.6% vs. 22.2; *p* = 0.042) (Table 2, Figure 4).

The inflammatory syndrome was greater in the first group than that in the second group [median maximum CRP level 289 vs. 211 vs. 151 mg/L (*p* < 0.001), neutrophil count 14,250 vs. 10,110 vs. 10,520/mm^3^ (*p* = 0.004), platelet count 539 vs. 305 vs. 375 G/L (*p* < 0.001) and albumin level 21 vs. 27 vs. 30 g/L (*p* < 0.001)]. The three groups did not significantly differ in the level of troponin (273 vs. 89 vs. 84 ng/L; *p* = 0.515) or brain natriuretic peptide (*p* = 0.361) (Table 3).

Chest x-ray abnormalities were more frequent, but not significantly, in the first group than those in the second and third groups (67.3% vs. 33.3% vs. 50%; *p* = 0.061) as was cardiac dysfunction on ultrasonography (67.2% vs. 52.8% vs. 76.9%; *p* = 0.204) (Table 3).

Corticosteroid treatment was less frequent in the first group than that in the second and third groups (47.6% vs. 97.8% vs. 85.2%; *p* < 0.001), but antibiotic therapy was more frequent (82.8% vs. 17.8% vs. 11.1%; *p* < 0.001) as was anticoagulant therapy (66.7% vs. 17.8% vs. 11.1%; *p* < 0.001) (Figure 3). Ventilatory support and inotropes were used more in the first group than those in the second and third groups. The mean time to corticosteroid administration was reduced in the second and third waves: 7.5 days in the first wave to 6.1 days in the second wave and 5.1 days in the third wave, but not significantly (*p* = 0.091). This observation was consistent with the decrease in diagnostic time previously observed. Moreover, we detected a weak positive correlation between time to corticosteroid administration and hospitalization duration (Spearman rho = 0.2566, *p* = 0.0178).

### 3.3. Severity factors

The length of hospitalization was weakly correlated with patient age (rho = 0.26, *p* = 0.0027), troponin level (rho = 0.44, *p* = 7.8 × 10^−7^), and CRP level (rho = 0.45, *p* = 6.45 × 10^−8^). We also detected a weak positive correlation between hospitalization duration and time to corticosteroid administration (rho = 0.2566, *p* = 0.0178).

Cardiac dysfunction was associated with symptoms of myocarditis [OR: 22.29; 95% CI: (7.25–68.53); *p* < 0.0001] and respiratory distress [OR: 2.56; 95% CI: (1.06–6.20); *p* = 0.039] (Table 4).

**Table 4:** Risk factors for the development of cardiac dysfunction.

	OR	95% CI	*p*
Sex female (%)	1.7812	(0.81–3.92)	0.169
Obesity (%)	1.8590	(0.47–7.30)	0.532
Overweight (%)	0.4200	(0.14–1.27)	0.146
Fever (%)	1.86	(0.11–30.68)	1.000
Abdominal pain (%)	1.21	(0.52–2.82]	0.670
Diarrhea (%)	1.77	(0.80–3.93)	0.223
Vomiting (%)	0.95	(0.43–2.10)	1.000
Cough (%)	0.79	(0.26–2.41)	0.773
Respiratory distress (%)	2.56	(1.06–6.20)	**0**.**039**
Myocarditis (%)	22.29	(7.25–68.53)	**<0**.**001**
Coronary dilatation (%)	1.25	(0.36–4.38)	1.000
Pericarditis (%)	0.83	(0.33–2.07)	0.814
Palmoplantar rash (%)	1.33	(0.47–3.81)	0.796
Edema extremities (%)	0.41	(0.13–1.34)	0.214
Desquamation (%)	1.45	(0.51–4.12)	0.613
Rash (%)	0.54	(0.23–1.27)	0.206
Conjunctivitis (%)	0.58	(0.25–1.34)	0.218
Lip cracking (%)	0.69	(0.31–1.54)	0.421
Headache (%)	1.37	(0.61–3.05)	0.544
Meningeal syndrome (%)	1.30	(0.50–3.34)	0.645
Rhinorrhea (%)	0.53	(0.07–3.92)	0.612
Pharyngitis (%)	0.64	(0.27–1.48)	0.382
Cervical adenopathy (%)	1.53	(0.58–4.08)	0.478
Arthralgia (%)	0.54	(0.03–8.82)	1.000
Myalgia (%)	0.42	(0.15–1.19)	0.106
Positive nasoph. COVID-19 PCR (%)	0.80	(0.35–1.82)	0.672
Leukocyturia (%)	1.94	(0.55–6.89)	0.345
EBV-positive blood PCR (%)	1.08	(0.21–5.45)	1.000
Positive lumbar puncture (%)	1.38	(0.38–5.01)	0.757
Abnormal chest x-ray (%)	2.57	(0.90–7.36)	0.109
Abnormal chest CT (%)	2.22	(0.40–12.29)	0.413

OR, odds ratio; 95% CI, 95% confidence interval; EBV, Epstein–Barr virus.

Bold values mean significant *p* < 0.05.

## 4. Discussion

Our prospective multicenter study thoroughly reviewed the symptoms and care of patients with MIS-C over the three first waves of the COVID-19 pandemic in France. Over the three waves, with the change in the management of MIS-C, children in the JIR cohort showed a less severe disease course and, in particular, a greater use of corticosteroids.

The age of the patients in our cohort was identical to that found in the literature, with a median age of 8.5 years (4, 10–13). The age at diagnosis of MIS-C was greater in the first than that in the second and third MIS-C groups, corresponding to the first three COVID-19 waves, respectively. COVID-19 vaccination did not start until July 2021 in France, so it cannot explain these differences. In contrast, this difference in age at diagnosis could reflect a better recognition of the pathology because it coincides with the dissemination of information on the existence of MIS-C in the specialized and non-specialized media, followed by the publication of the MIS-C criteria by the WHO. Indeed, many teams published in spring–summer 2020 to alert to new patients cared for in pediatric resuscitation and specificities of their condition (4, 5, 13, 14). A French time series found a major and rapid increase in Kawasaki disease cases of 497% in April 2020 (15), knowing that Kawasaki disease generally affects children under 5 years of age and has many similarities with MIS-C (4). Many patients with MIS-C were probably initially misdiagnosed as having Kawasaki disease, particularly children under 5 years of age. This overdiagnosis of Kawasaki disease in the under-5 age group could be the cause of an overestimation of the median age at diagnosis in the first wave rather than a true difference in age between the different waves.

Our results also favor a syndrome occurring in genetically predisposed children because 46% of the patients were of sub-Saharan African origin. This observation agreed with studies in the United States showing African Americans with a risk of MIS-C that was nine times greater than that for Caucasians (16–18).

Patients frequently had myocardial involvement from the first days of the disease. Most had myocardial edema, without myocarditis criteria, which agrees with a previous description (19). In contrast, obesity did not appear to be a risk factor in children.

We found a phenotypic and biological difference between the three MIS-C groups, with patients in the second and third waves being less symptomatic, with less inflammatory, and with fewer signs of severity, such as myocardial damage or respiratory distress, than those in patients in the first wave. Several hypotheses can explain these differences. The first is an improved recognition of MIS-C. Indeed, the mean diagnosis time was higher in the first and second groups than that in the third group. Although not significant, this shortening was necessarily associated with a more rapid identification of MIS-C cases presented to the emergency department and, therefore, earlier management in the second and third waves. This hypothesis could explain this phenotypic difference with less symptomatic patients at diagnosis and a more favorable evolution with fewer serious situations in the later waves. Other cohorts, notably in the United States, reported the same observation, with a decrease in mortality during the waves, which remained low at 1.2% of cases (20, 21). The second hypothesis is the appearance of SARS-CoV-2 variants, such as the Alpha strain, which began circulating in the autumn of 2020 and was dominant from March 2021 in France (22). The hypothesis of an Alpha variant generating fewer complications and a less intense inflammatory syndrome is possible. Of note, the Omicron variant, which has been predominant in France since the end of December 2021, was only detected for the first time after the end of the period of interest of this study. Because we could not access virus sequencing in routine practice at the time of infection, we do not have the necessary data to study this possible link.

Therapeutic management changed over the waves in our cohort. The addition of corticosteroids to IVIg therapy vs. IVIg therapy alone decreased the risk of cardiac dysfunction, treatment failure in the case of preexisting cardiac dysfunction, number of days with fever, and number of days in the intensive care unit (23, 24). Following these publications and French recommendations released in December 2020, changes in management occurred in French medical departments. As a result, we found an increase in the use of corticosteroids from the second group but also a decrease in the use of both ventilation supports and inotropes. The number of patients staying in intensive care units also decreased, as did the median hospitalization stay. Only 12 patients received biotherapy (10 with anti-IL1 agents and 2 with anti-IL6 agents); all had a favorable outcome. Of interest, the use of biotherapies did not increase over the waves. A French team showed that use of anakinra was associated with a rapid stabilization and recovery in seven patients with acute fulminant myocarditis in an MIS-C setting (25).

We looked for potential factors associated with cardiac dysfunction but found no clinical or biological factors associated with cardiac involvement, except for the presence of myocarditis.

There are multiple limitations to this study. Case reporting was voluntary. Therefore, the cases reported are mainly from main hospitals. This center effect may have influenced the disease severity of patients included and the adaptability of the teams to rapidly follow the changes in management protocols. This cohort would represent about 22.5% of MIS-C cases in France over the same period (26). Ongoing, more extensive recruitment would help in refining the data analyzed.

## 5. Conclusions

Over the first three epidemic waves of COVID-19 in France, cases of MIS-C exhibited a decrease in age, hospitalization duration, and myocardial damage. This report illustrates the impact of the treatment recommendations and early detection of MIS-C on outcomes.

## Data Availability

The original contributions presented in the study are included in the article, further inquiries can be directed to the corresponding author.
